# Positive Connectivity Predicts the Dynamic Intrinsic Topology of the Human Brain Network

**DOI:** 10.3389/fnsys.2018.00038

**Published:** 2018-08-30

**Authors:** Jingyu Qian, Ibai Diez, Laura Ortiz-Terán, Christian Bonadio, Thomas Liddell, Joaquin Goñi, Jorge Sepulcre

**Affiliations:** ^1^Department of Radiology, Gordon Center for Medical Imaging, Massachusetts General Hospital, Harvard Medical School, Harvard University, Boston, MA, United States; ^2^Neurotechnology Laboratory, Tecnalia Health Department, Derio, Spain; ^3^University of Exeter Medical School, Exeter University, Devon, United Kingdom; ^4^School of Industrial Engineering, Purdue University, West-Lafayette, IN, United States; ^5^Purdue Institute for Integrative Neuroscience, Purdue University, West-Lafayette, IN, United States; ^6^Weldon School of Biomedical Engineering, Purdue University, West-Lafayette, IN, United States; ^7^Athinoula A. Martinos Center for Biomedical Imaging, Charlestown, MA, United States

**Keywords:** functional connectivity MRI, positive connectivity, negative connectivity, stepwise functional connectivity, topological causality

## Abstract

Functional connectivity MRI (fcMRI) has become instrumental in facilitating research of human brain network organization in terms of coincident interactions between positive and negative synchronizations of large-scale neuronal systems. Although there is a common agreement concerning the interpretation of positive couplings between brain areas, a major debate has been made in disentangling the nature of negative connectivity patterns in terms of its emergence in several methodological approaches and its significance/meaning in specific neuropsychiatric diseases. It is still not clear what information the functional negative correlations or connectivity provides or how they relate to the positive connectivity. Through implementing stepwise functional connectivity (SFC) analysis and studying the causality of functional topological patterns, this study aims to shed light on the relationship between positive and negative connectivity in the human brain functional connectome. We found that the strength of negative correlations between voxel-pairs relates to their positive connectivity path-length. More importantly, our study describes how the spatio-temporal patterns of positive connectivity explain the evolving changes of negative connectivity over time, but not the other way around. This finding suggests that positive and negative connectivity do not display equivalent forces but shows that the positive connectivity has a dominant role in the overall human brain functional connectome. This phenomenon provides novel insights about the nature of positive and negative correlations in fcMRI and will potentially help new developments for neuroimaging biomarkers.

## Introduction

Functional connectivity MRI (fcMRI) is a powerful approach to investigate how areas of the human brain oscillate over time as a proxy of large-scale neuronal networks. Among several analytical approaches, a common fcMRI framework uses linear regressions to calculate couplings of spontaneous fluctuations of low frequency blood-oxygen-level-dependent (BOLD) signals between brain regions (Biswal et al., 1995). These signals can be mathematically transformed into graphs, where sets of nodes and edges/connections reflect linear correlations (Biswal et al., 1995; Hutchison et al., 2013), and whose arrangements define network features of the human brain. The combination of fcMRI with subsequent graph theoretical analyses has provided researchers with novel information about imaging biomarkers related to the (re)organization of cortical circuits in normal and in clinical conditions, such as neurodegenerative diseases (Hampel et al., 2010; Zhou et al., 2010; Albert et al., 2011; Prvulovic et al., 2011; Sepulcre et al., 2013, 2016).

Early descriptions of fcMRI patterns found that intrinsic brain activity can be classified as positive and negative connectivity depending on BOLD temporal relationships and the associated pairwise correlation values (Biswal et al., 1995; Fox et al., 2005). In this context, researchers have conceptualized the human brain as a functional system in which correlated and anti-correlated interactions evolve in time to form the basis of cognition and behavior (Fox et al., 2005). However, while the interpretation of positive correlations has been more intuitive, the nature and understanding of the negative correlations has created a passionate debate (Fox et al., 2007; Liang et al., 2012). It is well known that some fcMRI preprocessing pipelines may introduce an artificial increment of negative correlations that bias the analysis toward the anti-correlation effect (Weissenbacher et al., 2009) due to the regression of the mean brain (global) signal in order to diminish common sources of noise in all the voxels (Liu et al., 2017). Although this phenomenon does not question the existence of negative correlations *per se*, it gives rise to suspicions about the relevance of some findings involving anti-correlations, particularly when characterizing clinical population features. In addition, other groups have suggested that negative correlations should not be taken as real competing force on the functional brain but as an epiphenomenon of the network distance topology (Chen et al., 2011). For instance, if a pair of nodes in the brain network are distantly connected with many “relay stations” in the middle, then transmitting information from one to the other undergoes various disturbances such as noise and attenuation, that drives a functional delay/lag, resulting in a negative correlation between them. In this sense, the shortest path length along positive connections may explain the emergence of a negative correlation value between two brain regions in a more parsimonious manner, as previously suggested (Sporns et al., 2002; Achard, 2006; Chen et al., 2011). In summary, the understanding of negative connectivity in fcMRI remains somewhat enigmatic. Uncovering its nature is a pertinent task that will help researchers to comprehend functional connectivity findings and will impact in our future discoveries of clinical biomarkers.

In this study, we aim to investigate the relationship of positive and negative connectivity in fcMRI in order to elucidate their spatial-temporal interactions. As brain connectivity patterns are dynamic, and the structure of positive and negative network connections both evolve over time, we studied the temporal cortical changes of brain connectivity to evaluate the causal dependencies of positive and negative connections inside the network structure. We hypothesized that the dynamic changes of connectivity patterns will show the underlying nature of the positive and negative connectivity relationship. If negatives are true opposing forces, it is expected that, for instance, positive correlations will not explain the appearance in time of the negative ones, thus, a scenario in which sometimes positives will influence or induce negatives and other sometimes negatives will create changes in positives (evenly-explained outcome). Alternatively, if either positive or negative connectivity have a predominant role in the overall network dynamics, it is expected that one will induce the emergence of the other (positive-explaining-negative or negative-explaining-positive outcomes).

## Methods

### Overview

**Figure 1:**
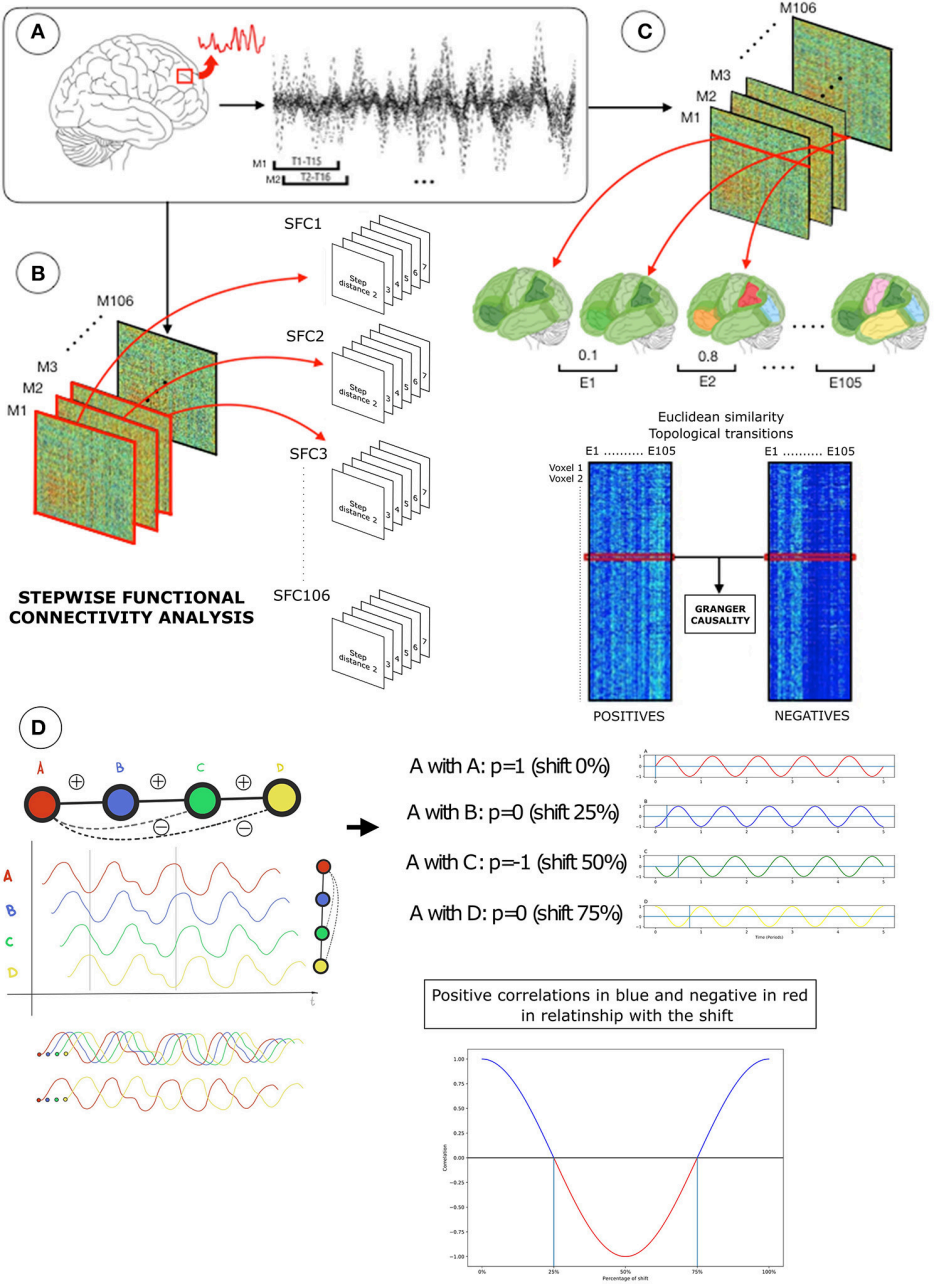
Diagram of the neuroimaging-processing pipeline in one subject. **(A)** Functional magnetic resonance images (fMRI) were transformed into functional connectivity processed BOLD signals (top-left). Using an overlapping sliding window approach (top-left; 15 time-points, 1 time-point lag), functional connectivity processed BOLD signals of voxels were used to create network association matrices based on Pearson R (Fisher transformed) correlations (top-right). **(B)** Network association matrices (M) were the input for two distinctive analytical strategies: (1) stepwise functional connectivity analysis (SFC) to compute optimal distances of the network (bottom-left) and (2) Granger causality analysis of Euclidian distance similarities over time (bottom-right). SFC analysis diagram represents the SFC matrices of positive correlations with the corresponding step distances from 2 to 7 in each network configuration (T1, T2 … T106) (bottom-left). Before the Granger causality analysis, each sliding window association matrix was separated into positive and negative correlations. **(C)** Then, each node-based network of positive or negative connections (rows or columns in the positive or negative association matrices; also known as star network) were used to compute the Euclidean distance E, where lower values represent a similar topology between networks and higher different topology between networks (displayed in similar or different colors). Euclidean distances between consecutive pairs of network configurations were computed within positive (bottom-right, left matrix in **C)** or negative connectivity (bottom-right, right matrix in **C)**, resulting in two matrices containing Euclidean distances of 1,902 voxels by 105 network transition pairs. Finally, a Granger test is applied to the Euclidean distance of positive and negative connectivity time transitions (red lines in Euclidean distance matrices; bottom-right) to determinate if positive-over-negative or negative-over-positive connectivity changes dominate network landscape. In **(D)** transitions from positive to negative connectivity are displayed for illustration purposes. Assuming a theoretical BOLD time series (red wave), it is possible to observe the arise of negative correlations due to specific lags and graph connectivity distances.

Figure 1 shows the major sections of our working pipeline. (1) First, we used a sliding window approach (Leonardi and Van De Ville, 2015) and stepwise functional connectivity analysis (SFC) (Sepulcre et al., 2012) to investigate [and confirm previously reported findings (Chen et al., 2011)] the potential relationship between the connectivity distance and strength of negative correlation between pairs of brain regions. All connectivity relationships between optimal step distances and topological patterns were evaluated at the voxel-wise level. (2) Second, we used an overlapping sliding window approach (Hutchison et al., 2013; Allen et al., 2014; Leonardi and Van De Ville, 2015), voxel-wise Euclidean-distance-based causality analysis to investigate the temporal dependencies between the dynamic changes of the positive and negative connectivity cortical patterns.

### Participants

Fifth-one healthy subjects from Brain Genomic Superstruct Project (Holmes et al., 2015) were included in the study. A set of 11 subjects (age 37.8 ± 13.6; 5 male and 6 female) was used as the discovery dataset and an additional 40 individuals from an independent dataset (age 32.9 ±11.7; 20 male and 20 female) was used as a replication dataset. Harvard University and Partners Healthcare institutional review boards (IRBs) approved the Brain Genomic Superstruct Project study. Participants provided written informed consent in accordance with Helsinki Declaration and guidelines set by IRBs of Harvard University or Partners Healthcare.

### Imaging acquisition procedures and functional connectivity preprocessing

Subjects were scanned on a 3 Tesla (ACHIEVA 3.0T TX, Philips) MRI scanner using an 8-channel phased-array head coil. High-resolution 3D T1-weighted magnetization prepared rapid acquisition gradient echo (T1W 3D TFE SENSE) images were acquired for anatomic reference (TR = 7.6 ms, TE = 3.5 ms, FA = 7 u, 1.0 mm isotropic voxels). Functional data was acquired in 3 runs using an echo planar imaging (EPI) pulse sequence sensitive to BOLD contrast (TR = 3,000 ms, TE = 30 ms, FA = 90 u, 3.0 mm isotropic voxels), each run lasted 6 min 12 s (hence producing 124 time-points or observations per run). In both runs the subjects were asked to lie still with their eyes open. Subject's head was fixed as much as possible using a pillow and foams, and earplugs were provided to reduce the scanner noise.

The fMRI BOLD (blood oxygen level-dependent) signal was optimally preprocessed for our r-fcMRI analysis according to Biswal et al. (1995). An optimized functional connectivity magnetic resonance imaging (fcMRI) protocol (Van Dijk et al., 2010), extending the approach developed by Biswal et al., was used in the preprocessing steps (DPABI/DPARSF toolbox). We used SPM12 (Wellcome Department of Cognitive Neurology, University College of London, London, UK, http://www.fil.ion.ucl.ac.uk/spm) for imaging preprocessing and normalization of the anatomical T1-weighted MRI images. The original fMRI data first went through typical steps such as the removal of the first four volumes, motion correction and normalization to the MNI atlas space. We applied a band-pass filtering (0.01–0.08 Hz) to diminish the effect of low-frequency drift and high-frequency noise and a scrubbing of image volumes with excess head motion [frame displacement >0.5 mm (Jenkinson et al., 2002)] through interpolation to transform fMRI data into desired r-fcMRI data. It has been previously reported that the global signal regression may induce artificial negative correlations (Murphy et al., 2009; Saad et al., 2012), thus, given the focus of our work in understanding positive and negative functional connectivity, we did not regress out the global signal but the averaged signal from ventricle and white matter and 24 head motion parameters. Finally, the data was down-sampled at 8 mm isotropic voxel size to study the high dimensional data without computational limitations. Please note that this work was performed at the voxel-level. Thus, brain regions are equivalent to individual voxels. All our analyses were conducted on Matlab R2015b (Mathworks Inc., Natick, MA).

### Sliding window approach

Conventional fcMRI approaches derive connectivity information from the whole length of the BOLD time series and results in a stationary, time-averaged brain network graph. However, brain network dynamic changes occur at many temporal scales and other strategies have been used to take full advantage of the temporal information contained in the fcMRI data. The sliding-window approach extracts the dynamic interactions between brain areas by using a time moving-window along the BOLD time-series (Hutchison et al., 2013; Allen et al., 2014; Leonardi and Van De Ville, 2015). Following that strategy, we use an overlapping time-moving window *w[t]*_*i*_ of length 15 on the time series (15 time-points per window, corresponding to 45 s) to calculate Pearson's cross-correlations (R) (Figure 1A):

$$w{\left[t\right]}_{i}=\{\begin{array}{l} 1,\;\left(i-1\right)n+1\leq t\leq \left(i-1\right)n+m\\ 0,\;otherwise \end{array}$$

Where *i* denotes the sliding window position, *t* states the specific time point, *n*, being the sliding offset, indicates how many time points the window has shifted along the time axis, and *m* specifies the window size. For example, if the offset *n* = *1*, then time points 1–15 will be included in the window. Shorter window length might provide higher temporal resolution of transient changes, but will lack precision to estimate correlation coefficients. On the contrary, longer window length might improve precision, but the result will tend toward the time-averaged solution (Hutchison et al., 2013). Our overlapping criterion was to let *n* increments 1 at a time, in order to obtain smooth transitions between network states. We used a brain gray matter mask (at MNI space) containing 1,902 voxels to extract the BOLD time series (120 time-points per run) and applied the above-mentioned sliding window approach. This step generated 106 dynamical functional connectivity matrices per run, each 1,902 × 1,902 in size. Finally, we applied a *Fisher* transformation to all correlation coefficients of association matrices for variance stabilization (Fisher, 1915).

### Stepwise functional connectivity analysis

As previously reported, the strength of negative correlations between a pair of brain network nodes–or brain voxels in this study- may relate to their link-step distance (also known as path-length or network geodesic distance). In order to confirm this hypothesis, first, we used stepwise functional connectivity analysis (Sepulcre et al., 2012) to compute the optimal (or minimal) distance between node pairs in all dynamical functional connectivity graphs separately per subject (Figure 1B). Particularly, for given nodes ***i*** and ***j*** and step distance ***l***, we calculated the weighted degree of SFC as the weight of positive paths (paths with a positive *Fisher* transformed correlation value) connecting ***i*** and ***j*** that have length ***l***. In this sense, a larger SFC degree under the step distance ***l*** indicates a main connection stream, while a smaller degree means less connectivity paths. Importantly, the original description of SFC analysis was developed using seeds of interest and binary data to reveal connectivity transitions across specific systems (Sepulcre et al., 2012). Here, we did not use any seed or region of interest but computed the SFC for all possible voxels in the brain in weighted data (non-binary). Each SFC matrix *A*_*l*_ of size m-by-m can be recursively represented as follows:

$$A_{l}\left(i,j\right)=\{\begin{array}{c} A\left(i,j\right),\wedge l=1\\ \sum_{k=1}^{m}A_{l-1}\left(i,k\right)A\left(k,j\right),\wedge l\geq 2 \end{array}$$

Here *A*_*l*_ is the functional connectivity matrix having the step distance of ***l***, and *A* is the correlation matrix after the sliding window approach and *Fisher* transformation. Since we were interested in investigating how the negative strength between pairs relates to their link-step distances of positive correlation sign, the step distance of 1 can be excluded from the SFC analysis because, by definition, a pair of nodes in step one (direct connectivity) cannot display a simultaneous negative correlation value if it has a positive connection already. Therefore, we calculated SFC from step distance 2–7 (based on the stable state of functional connectivity data (Sepulcre et al., 2012), step 7 is the maximum step distance or longest path that can be found). This step generated six SFC matrices (one for each distance) × 106 time frames per run (Figure 1B). Then, for each connectivity step, we normalized all SFC matrices between 0 and 1 (each SFC value minus the minimum SFC value divided by the maximum SFC value minus the minimum SFC value). This step does not change the final distribution of values but makes them comparable across all step distances. Then, we compared all normalized SFC matrices element-wise, found the maximum corresponding SFC degree value, and assigned their corresponding distance step matrix that belongs to as the optimal distance (OD in equation) value (from 2 to 7).

$$OD\left(i,j\right)=argmax_{l}\;A_{l}(i,j)$$

Later, we investigate the association between the average optimal distance values and average negative correlation values of all pair of nodes across all time points.

### Cortical spatial similarity and dynamic topological causality

The general aim of this study was to investigate the phenomenon of negative connectivity and its significance in the network structure of fcMRI data. If negative and positive connections represent opposing forces of equal dominance in brain graphs, it is expected that at times, one may control the other, but neither will consistently predict their appearance across time. On the contrary, if positive and negative connectivity produce temporal dependencies between each other, the dynamic changes of one of them will significantly create dynamic connectivity changes in the other one. Thus, assessing the spatial-temporal causality between configurations of the positive and negative networks is key to understand functional network arrangements in the human brain. In order to accomplish this aim, we first developed a strategy to detect the temporal transitions of the positive and negative networks in the cortical mantle. We split each of the original dynamical functional connectivity matrices into two matrices, one containing the positive connections and the other one containing the negative connections (Figure 1C). Then, we used Euclidean distance to identify the similarity or dissimilarity of network configuration over time between each consecutive pair of networks at the node level (see cortical maps in Figure 1C). That is, we computed the Euclidean distance between pairs of networks across time by taking into account, separately, the positive or negative connectivity patterns of each node. Thus, for each node/voxel in the human brain, we obtained its network similarity scores over time for both the negative- and positive-based connectivity networks (Euclidean distance matrices of positive and negative connectivity transitions; Figure 1C). Each node's temporal Euclidean distance between two consecutive network configurations *t*_1_ and *t*_2_ is defined by the following equation:

$$D_{i,t_{1}t_{2}}=\sqrt{\sum_{j=1}^{n}{\left(r_{t_{1}}(i,j)-r_{t_{2}}(i,j)\right)}^{2}}$$

Where *D* is the Euclidean distance, *n* is the total number of nodes considered, *r*_*i*_*t*__1__ and *r*_*i*_*t*__2__ represents the R values that node has with another node, in two consecutive time points. For each voxel, we calculated the temporal Euclidean distance fluctuations of both the network of positive and negative connectivity separately. Larger *D* means the node is experiencing dramatic change in its self-network structure or connectivity pattern, and smaller *D* indicates minor transitions between two time points. Thus, the fluctuation of Euclidean similarity scores across time helps us to apprehend how each node's network structure resembles itself over time. More importantly, it provides means to compare transitions states of networks at the voxel-level, and study the causal inferences between them.

Finally, we applied a Granger causality analysis (Granger, 1969) to the network similarity scores across all Euclidean distance changes to investigate temporal causalities between the positive and negative connectivity networks (Euclidean distance matrices of positive and negative connectivity transitions; Figure 1C). Our Granger causality analysis is based on linear regression assumptions (Roebroeck et al., 2005) (Granger causality open-source code can be found at http://www.mathworks.com/matlabcentral/fileexchange/25467-granger-causality-test). We used the two Euclidean distance transition series of the positive and negative connectivity as the input and hypothesis testing values (F-statistics) as the output. Of note, we did not use Granger causality to test the temporal relationships between BOLD signal fluctuations directly, but rather to investigate the cortical topological transitions (or Euclidean distances between network configurations) that positive and negative correlation networks develop over time and how they affect each other (positive-explaining-negative, negative-explaining-positive, or evenly-explained in the case of none significant findings). Specifically, if adding past values of Euclidean distances X (rather than simply using the past values of Euclidean distances Y) better predicts the current values of Euclidean distances Y, then it implies that Euclidean distances X is series Y's granger cause. The hypothesis of “X causing Y” can be formally described as a regression model:

$$y\left(t\right)=\sum_{m=1}^{p}a_{m}y\left(t-m\right)+\sum_{n=1}^{q}b_{n}x\left(t-n\right)+\varepsilon +c$$

where the first item measures the effect of past self-values, the second item measure the effect of past cause values, and the third and forth items are the error and a constant. We used a Bayesian criterion (http://www.mathworks.com/matlabcentral/fileexchange/25467-granger-causality-test) to identify the optimal lag between the two connectivity cortical transitions (from 1 to 5 consecutive network topologies in the cerebral cortex). We assessed two voxel-level statistical thresholds, (1) an α-level < 0.05, and (2) false discovery rate (FDR) at a q-level of 0.05 to all F-statistic values.

### Visualization

For visualization purposes, we used the F-statistic values from our two testing hypotheses (positive-explaining-negative and negative-explaining-positive) and performed a subtraction between cortical maps at the voxel-level in order to highlight their spatial predominance. We used Caret software (PALS surface (PALS-B12) (Van Essen, 2005); interpolated algorithm and multi-fiducial mapping) as the final cortical space visualization tool.

## Results

### Correlation strength and path-length are related in positive and negative connectivity

**Figure 2:**
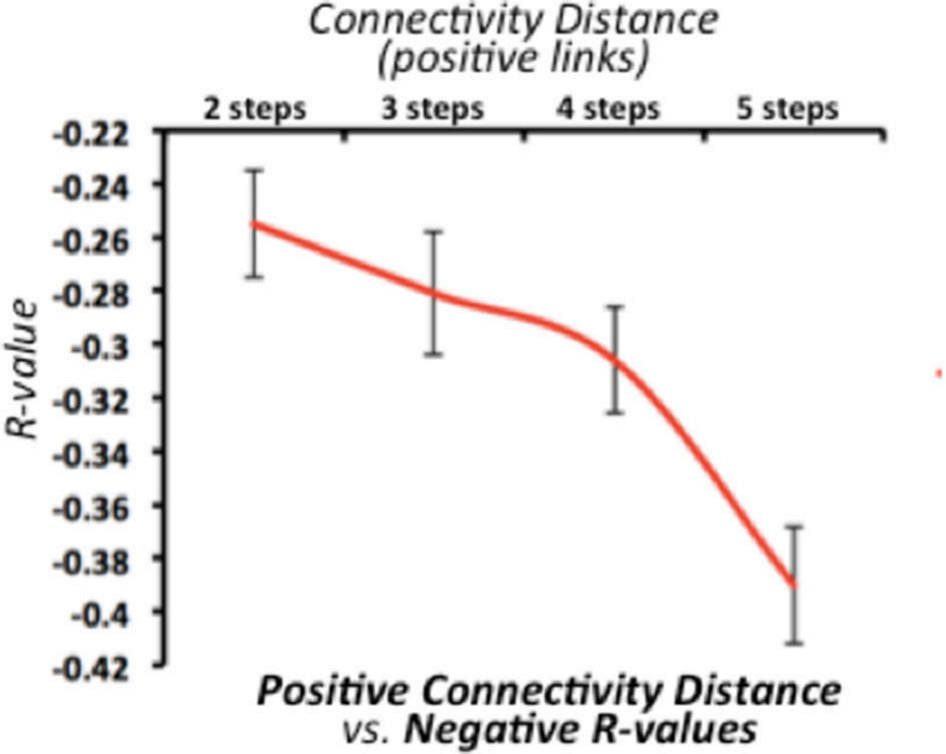
Relationship between stepwise functional connectivity (SFC) optimal distance and average negative correlations in the discovery dataset. X-axis represents the SFC optimal distance for all positive connections. X-axis represents the average negative correlation value. Error bars indicate the standard deviation across all subjects. Of note, line graph starts in two steps -as negative *R*-values are incomputable if a positive functional connectivity is already established.

We found that the strength of negative connectivity between pair of nodes directly relates to their path-length through positive connections (Figure 2). The average optimal distance ranges from 2 to 5 steps. Thus, we observed a decreasing relationship between the average negative *R*-values and the SFC optimal distance, in which strong negative correlations (*r* > −0.35) relate to large connectivity routes through positive connections along the functional connectivity matrix. This result confirms that both connectivity types have an interdependent nature regarding the overall network topology complex. However, this finding does not provide insights about whether one type of connectivity causes the other, or if one has a leading role in the dynamic functional connectivity changes.

### Dynamic connectivity patterns of positive connections predicts negative connections

**Figure 3:**
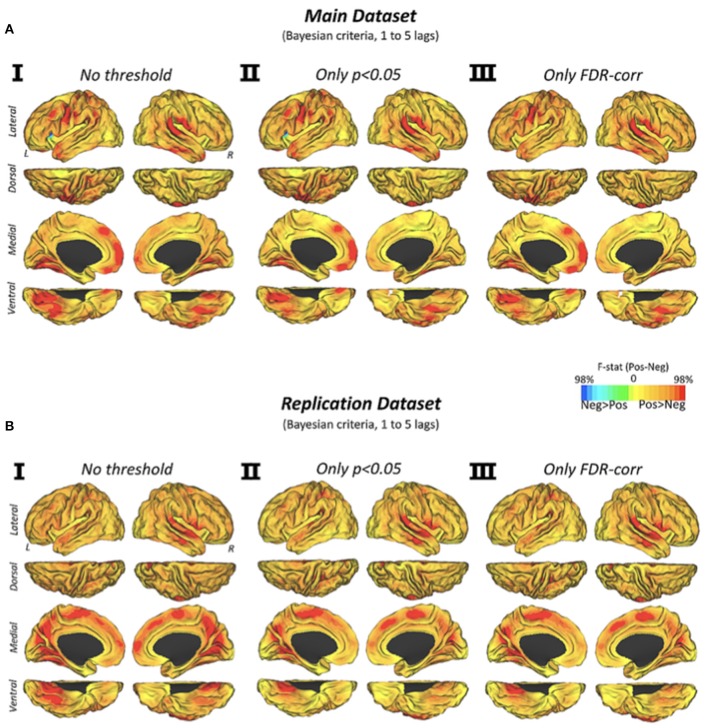
Cortical Maps of Granger causality between positive and negative network configurations (via Euclidean distance similarities) over time in the discovery and replication datasets. Cortical maps show the Granger causality findings from two testing hypotheses (positive-explaining-negative and negative-explaining-positive connectivity) in three statistical conditions **(I)** no statistical threshold, **(II)** *p*-value < 0.05, and **(III)** *p*-value corrected by an FDR approach with q level at 0.05. Color scale shows the subtraction of F-statistics with a normalized intensity of positive and negative values using a 0–98% transformation.

The dynamic transitions of connectivity over time showed that positive connectivity significantly precedes the appearance of new negative connectivity configurations (Figure 3A). We found that the vast majority of the human brain displays this predominant causality pattern, particularly primary cortices such as visual, auditory and somatomotor cortex, and temporal, frontal midline or perisylvian areas. Similar findings were obtained by using different statistical strategies (Figure 3AI, *p* < 0.05, discovery dataset; Figure 3AII, FDR-corrected, discovery dataset; Figure 3AIII, *p* < 0.05, replication dataset; Figure 3B, FDR-corrected, replication dataset).

## Discussion

In the past there have been various efforts trying to better understand negative connectivity fcMRI data. Our understandings of negative correlations have evolved over the recent years, from concerns about possibly being induced by preprocessing steps (Murphy et al., 2009) to enthusiasms about its potential as disease biomarker (Whitfield-Gabrieli and Ford, 2012). However, several groups have suggested that negative connectivity may be an emergence phenomenon in the fcMRI data, in which they do not represent a true competing force against the positive connectivity but a result of the network complexity organization. For instance, Chen et al. support that negative connectivity may be just the result of long path-lengths between brain regions (Chen et al., 2011), a finding that is confirmed in the present study. Murphy et al. suggest a mechanism whereby artifactual anti-correlations could arise after global regression from a delay correlation between systems (Murphy et al., 2009). Moreover, Goelman et al. used both human and rodent data to assess the effect that time-lag has on positive correlations and negative correlations, and used cerebral blood volume and flow to explain the potential emergence of negative correlations (Goelman et al., 2014).

The human brain is constantly engaged in multi-scaled and synchronized neuronal couplings. fcMRI is used as a proxy to understand these neuronal couplings at the large-scale and network level. Although positive and negative connectivity has been described with fcMRI, researchers are still wondering whether they are truly opposite forces or whether they have to be considered signals of equal value to investigate the human brain functional connectome. It seems natural that positive couplings between cortical regions arise to support local and distant communications (Sepulcre et al., 2010; Cabral et al., 2011). However, at present, we do not know whether positive and negative connectivity are mutually necessary for this function. For instance, from a parsimonious viewpoint, it is possible to support that simple de-synchronization phenomenon among positively connected regions may be sufficient to discontinue functional connections, making unnecessary the existence of negative forces. In the past, many research groups have reported significant associations between negative connectivity and a wide range of behavioral, neuropsychological and clinical scores, particularly related to neuropsychiatric disorders. However, these findings, along with related interpretations, have been described without a solid knowledge about the nature of the negative connectivity and its relationship with the positive ones. To clarify this point, in this study, we proposed a new strategy to elucidate not just the network distance relationships -which does not resolve the main uncertainties about the nature of negative connectivity- but to reveal the dynamic and causal dependencies of positive and negative connectivity in the human brain. First, we found that negative correlations between brain voxels are indeed related to their distance in the graph structure. Stronger negative correlations between nodes correspond to longer SFC optimal distance on the positive paths, acknowledging that negative correlation may arise due to an epiphenomenon of functional connections when taking into account the whole complexity of the graph. But more importantly, using Granger causality analysis on the evolving positive and negative connectivity patterns, we discovered that each node's positive connectivity changes precede negative connectivity changes in a higher degree that the reverse pattern. As shown in the cortical visualization (see Figure 3 and Supplementary Figure 1), this is a voxel-wise and brain-wide phenomenon, which occurs, particularly, in primary regions such as auditory, somatomotor, and visual areas. Thus, it is possible to speculate that regions with strong connectivity of the positive sign, such as those involved in primary regions, may be more prominent in predicting their topology changes over time due to its highly-local organized connectivity patterns. On contrast, regions with moderate or low levels of positive correlations may be more predispose to switch between synchronized and de-synchronized relationships with other brain regions, making them more unpredictable to drive future connectivity. Of note, negative connectivity, regardless of their theoretical strength, only marginally predicts the topology of positive connections.

In conclusion, the findings of our study extend our interpretation of negative connectivity in fcMRI data. They support that a positive-explaining-negative connectivity is the most relevant scenario to explain the interactions between these two hallmarks in fcMRI. Moreover, it favors the idea of choosing positive connectivity alone as selection criteria in fcMRI approaches, as they have a primary and central role in the overall organization of functional connectivity brain graphs. Finally, we believe that the concept of relative distance of connectivity between nodes, rather than just positive and negative connectivity, should be taken into account as a better proxy for network relationships. Further work will lie in the area of interaction of positive/negative networks during task state, as well as its clinical application regarding neuropsychiatric disorders in order to fully characterize the positive and negative network interactions.

## Author contributions

JQ, JG, and JS design and conduct of the study. JQ, ID, LO-T, CB, TL, JG, and JS analysis and preparation, review, or approval of the manuscript, decision to submit the manuscript for publication. JQ, JG, and JS interpretation of the data.

### Conflict of interest statement

The authors declare that the research was conducted in the absence of any commercial or financial relationships that could be construed as a potential conflict of interest.

## Supplementary material

Supplementary Figure 1 Cortical maps of Granger causality between positive and negative network configurations (via Euclidean distance similarities) over time in a second replication dataset (*N* = 45) for sliding window sizes of 30 (10 time points), 45 (15 time points), and 60 (20 time points) seconds. Color scale shows the subtraction of F-statistics with a normalized intensity of positive and negative values using a 0–98% transformation.
