# Supplementary figures and images for: Positive Connectivity Predicts the Dynamic Intrinsic Topology of the Human Brain Network
Source: Front Syst Neurosci. 2018 Aug 30;12:38. doi: 10.3389/fnsys.2018.00038 (PMC6125351; doi:10.3389/fnsys.2018.00038)

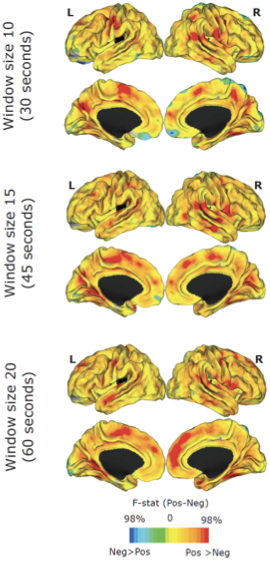

Supplement: Supplementary Figure 1 — Cortical maps of Granger causality between positive and negative network configurations (via Euclidean distance similarities) over time in a second replication dataset (N = 45) for sliding window sizes of 30 (10 time points), 45 (15 time points), and 60 (20 time points) seconds. Color scale shows the subtraction of F-statistics with a normalized intensity of positive and negative values using a 0–98% transformation. [file Image_1.TIFF]
